# Neurological diversity and generalizability: building on AMPA receptor imaging advances in long COVID

**DOI:** 10.1093/braincomms/fcaf499

**Published:** 2025-12-16

**Authors:** Thorsten Rudroff

**Affiliations:** Turku PET Centre, University of Turku and Turku University Hospital, Turku, Finland

## Abstract

This scientific commentary refers to ‘Systemic increase of AMPA receptors associated with cognitive impairment of long COVID’ by Fujimoto *et al.* (https://doi.org/10.1093/braincomms/fcaf337)

**This scientific commentary refers to ‘Systemic increase of AMPA receptors associated with cognitive impairment of long COVID’ by Fujimoto *et al.* (https://doi.org/10.1093/braincomms/fcaf337)**

Fujimoto *et al*.^1^ have achieved a remarkable breakthrough in understanding long COVID pathophysiology. Their study represents the first application of [¹¹C]K-2 PET imaging to cognitive impairment in long COVID (Cog-LC), revealing systemic AMPA receptor upregulation across multiple brain regions. The methodological rigour—careful patient selection, comprehensive exclusion of confounding conditions, sophisticated PET imaging and cytokine correlation—is exemplary. The diagnostic accuracy (100% sensitivity, 91.2% specificity) represents substantial progress for a condition affecting 400 million people globally with limited objective biomarkers.^2^ The identification of AMPA receptor upregulation as a therapeutic target and correlation with cytokine profiles (TNFSF12, CCL2) provides mechanistic insights that could explain variable anti-inflammatory treatment efficacy.

Building on these contributions, this commentary examines how demographic factors may influence global translation of these findings. This discussion aims not to diminish the study's significance but to strengthen its clinical application by identifying opportunities for validation across the diverse populations affected by long COVID worldwide. Throughout this commentary, I use ‘neurological diversity’ to refer to population-level variation in brain structure and function across demographic groups—reflecting genetic differences, environmental exposures and social determinants of health. This is distinct from ‘neurodiversity’, which describes variation in neurodevelopmental conditions such as autism, ADHD and dyslexia.

## Demographic representation: a field-wide challenge

The Fujimoto study^1^ appropriately matched age and sex within their Japanese cohort recruited from Yokohama City University. The homogeneous geographic and ancestral origin reflects a common pattern across major neuroimaging studies rather than a unique limitation.

Western neuroimaging research faces identical challenges. UK Biobank neuroimaging shows 94.6% White representation despite serving a more diverse nation.^3^ The Alzheimer's Disease Neuroimaging Initiative (ADNI) demonstrates less than 5% Black/African American participation despite this population's disproportionately higher dementia risk.^4^ Similar homogeneity characterizes European and North American cohorts.

The concern is not that the study was conducted in Japan—regional research excellence is essential—but that findings from any demographically homogeneous cohort require validation before extrapolation to populations not represented in the source data. This applies equally to Japanese-only, White-predominant or other single-ancestry studies.

## Why population diversity matters: biological mechanisms

AMPA receptor biology provides specific reasons why population-level variation could affect the Fujimoto findings’ generalizability. This represents scientific opportunity rather than criticism—understanding these mechanisms enhances our ability to predict how findings translate across populations.

**Genetic variation** in glutamate receptor subunit genes (GRIA1-4) demonstrates significant population stratification.^5^ The regulatory proteins controlling AMPA receptor trafficking to synapses—which the [¹¹C]K-2 tracer specifically measures—show similar variation. These genetic differences do not invalidate the Fujimoto findings but suggest baseline AMPA receptor density may differ across populations, affecting what constitutes ‘elevated’ levels. If one population's genetic variants produce naturally higher AMPA receptor expression, the same absolute PET signal might represent normal variation rather than pathological elevation.

**Environmental factors** including nutrition, chronic stress, pathogen exposure, and developmental context vary systematically across populations with different social determinants of health. Chronic stress exposure, which differs substantially between populations experiencing different socioeconomic conditions, affects glutamatergic system function through glucocorticoid-mediated mechanisms. Nutritional factors influence glutamate metabolism and AMPA receptor regulation. These environmental influences create epigenetic modifications that alter AMPA receptor expression patterns, potentially leading to different baseline densities across populations.^6,7^

**Immune-glutamate interactions** add another layer of complexity. The Fujimoto study^1^ elegantly demonstrates positive correlations between AMPA receptor density and plasma TNFSF12, with negative correlations for CCL2. However, baseline levels and inflammatory response patterns for these immune markers demonstrate known population-level variation.^8^ A recent study using [¹⁸F]FEPPA PET showed persistent neuroinflammation after COVID-19, with patterns suggesting sustained cytokine elevation drives ongoing pathology.^9^ If baseline inflammatory states differ across populations—as evidence suggests they do due to differential pathogen exposure, nutritional status and chronic stress—the association between cytokines and AMPAR upregulation may show population-specific patterns.

These biological mechanisms converge to suggest that what appears as ‘pathological’ AMPA receptor elevation when compared to one population's baseline might represent normal variation when compared to another population's genetically and environmentally determined baseline. This does not mean the Fujimoto findings are wrong—rather, it suggests the interpretation framework may need population-specific calibration.

## Evidence from other diagnostic algorithms

Experience with other neuroimaging AI systems demonstrates that demographic homogeneity in training data predicts performance disparities in clinical deployment. This evidence base helps anticipate validation needs for the Fujimoto diagnostic framework.

Seizure detection algorithms show 27% lower sensitivity in adolescent females compared to adult males when trained predominantly on adult male data.^10^ Dementia screening systems exhibit doubled false-positive rates in Black participants compared to White participants, reflecting training dataset biases.^11^ Most directly relevant: brain age prediction models—using the same analytical approaches as the Fujimoto study—show systematic errors when applied across populations.

A recent *Nature Communications* study documented COVID-19-accelerated brain ageing with largest effects in socioeconomically deprived individuals (up to 5.8 months additional predicted ageing).^12^ Critically, these models were trained on UK Biobank data representing predominantly White, educated, wealthy populations. The disparities raise questions about whether AI systems capture genuine neurological pathology or instead mistake neurological consequences of social inequality for inherent brain differences. When training datasets systematically exclude populations experiencing social disadvantage, resulting systems may pathologize normal neurological variation in those populations.

The Fujimoto algorithm achieved exceptional accuracy (100% sensitivity, 91.2% specificity) within the Japanese cohort. However, validation in other populations is needed to determine whether this accuracy translates to demographically diverse long COVID patients worldwide. Consider the scenario: if baseline AMPA receptor density differs across populations due to genetic or environmental factors, a diagnostic cut-off optimized for Japanese participants might systematically misclassify patients from other populations—producing false negatives in populations with naturally higher baselines or false positives in populations with lower baselines.

## Enhancing clinical translation: constructive pathways

Several actionable approaches could enhance clinical translation.

Multi-site replication studies: Partner with groups in diverse geographic regions to validate AMPAR upregulation. Sites in Africa, Europe, Latin America and other Asian countries would test whether findings represent universal COVID-19 pathophysiology or population-specific variation.

**Stratified analysis**: Within Japan, analyses by socioeconomic factors, urban/rural residence or occupational exposure might reveal whether AMPAR patterns vary with social determinants.

**Cytokine-AMPAR validation**: Test correlations in existing biobanks from diverse populations to determine universal replication.

**Longitudinal validation**: Track whether AMPAR density predicts clinical trajectory consistently across diverse populations.

**For therapeutic translation**: AMPA receptor antagonist trials should include diverse populations from inception, monitor for population-specific efficacy or safety signals and consider dose adjustments if baseline AMPAR density varies.

**For diagnostic development**: Before deployment, algorithms should undergo external validation in multiple independent cohorts from diverse populations, report performance metrics stratified by demographics, establish population-specific normative data and develop recalibration methods if performance varies.

## Broader context: population diversity as scientific necessity

When studies systematically exclude 85–95% of global population diversity, resulting diagnostic frameworks face fundamental validity threats regardless of technical excellence. Including diverse populations in research is not advocacy—it is a core requirement for scientific rigour and generalizability. Valid biomarker research must establish whether findings represent universal biology or population-specific patterns.

The Fujimoto study^1^ exemplifies high-quality regional research that requires international collaboration for global application. This represents opportunity rather than limitation—the findings provide foundation for multinational consortia to validate AMPAR imaging as a universal long COVID biomarker. Such collaborations could establish whether Japanese, European, African and Latin American populations show similar AMPAR responses to COVID-19 or whether population-specific patterns necessitate adapted diagnostic thresholds.

Scientific rigour demands that research acknowledges population-level neurobiological variation as biological reality. There is no single ‘normal’ brain—variation across populations reflects genetic diversity, environmental exposures, developmental contexts and social determinants of health.^13^ Valid diagnostic frameworks must incorporate this diversity rather than treating it as statistical noise. Systematic exclusion of populations undermines external validity and limits clinical translation.

## Conclusion

Fujimoto *et al*.^1^ have made exceptional contributions to understanding long COVID neuropathology through innovative AMPA receptor imaging, revealing molecular mechanisms underlying cognitive impairment, identifying therapeutic targets and demonstrating impressive diagnostic accuracy.

Responsible clinical translation requires extending findings to demographically diverse global populations. Challenges identified apply equally to Western research—reflecting field-wide opportunities for improvement rather than limitations unique to any study. The framework proposed here—assessing generalizability limitations, identifying biological mechanisms of population variation and establishing validation requirements—applies broadly to neuroimaging biomarker research in demographically homogeneous cohorts, whether Asian, European or North American. International collaborations building on the Fujimoto foundation could establish whether AMPAR upregulation represents universal COVID-19 pathophysiology or shows population-specific patterns requiring adapted diagnostic and therapeutic approaches.

The COVID-19 pandemic has disproportionately affected socioeconomically disadvantaged populations worldwide. Long COVID research must not replicate these inequities by developing frameworks that work primarily for populations represented in research cohorts. Realizing this work's full clinical potential requires validation acknowledging that neurological diversity represents fundamental biology rather than methodological inconvenience.

## Data Availability

There are no new data associated with this article.
